# Multidimensional analysis of the impact of Gemmatimonas, Rhodothermus, and Sutterella on drug and treatment response in colorectal cancer

**DOI:** 10.3389/fcimb.2024.1457461

**Published:** 2024-10-08

**Authors:** Shaowen Jin, Wa Zhong, Bo Li, Kaimei Wang, Dongming Lai

**Affiliations:** ^1^ Department of Gastrointestinal Surgery, Sun Yat-Sen Memorial Hospital, Sun Yat-Sen University, Guangzhou, China; ^2^ Department of Orthopedics, Beijing Luhe Hospital, Capital Medical University, Beijing, China; ^3^ Department of Pediatrics, Sun Yat-Sen Memorial Hospital, Sun Yat-Sen University, Guangzhou, China; ^4^ Department of Gastrointestinal Surgery, Shenshan Medical Center, Sun Yat-Sen Memorial Hospital, Sun Yat-Sen University, Shanwei, China

**Keywords:** colorectal cancer, microbiome, immunotherapy, drug sensitivity, risk score model NKG7, GNLY, KLRD1

## Abstract

**Background:**

Colorectal cancer is the third most prevalent cancer across the globe. Despite a diversity of treatment methods, the recurrence and mortality rates of the disease remain high. Recent studies have revealed a close association of the gut microbiota with the occurrence, development, treatment response, and prognosis of colorectal cancer.

**Objective:**

This study aims to integrate transcriptome and microbiome data to identify colorectal cancer subtypes associated with different gut microbiota and evaluate their roles in patient survival prognosis, tumor microenvironment (TME), and drug treatment response.

**Methods:**

An integrated analysis of microbiome data was conducted on samples of colorectal cancer from public databases. Based on this, two tumor subtypes (C1 and C2) closely associated with patient survival prognosis were identified and a risk score model was constructed. The survival status, clinical parameters, immune scores, and other features were analyzed in-depth, and the sensitivity of various potential drugs was examined.

**Results:**

A thorough examination of microbiome information obtained from colorectal cancer patients led to the identification of two primary tumor clusters (C1 and C2), exhibiting notable variations in survival outcomes. Patients with the C1 subtype were closely associated with better prognosis, while those with the C2 subtype had higher gut microbial richness and poorer survival prognosis. A predictive model utilizing the microbiome data was developed to accurately forecast the survival outcome of patients with colorectal cancer. The TME scores provided a biological basis for risk assessment in high-risk (similar to the C2 subtype) patient cohorts. Evaluation of the sensitivity of different subtypes to various potential drugs, indicated the critical importance of personalized treatment. Further analysis showed good potential of the developed risk-scoring model in predicting immune checkpoint functions and treatment response of patients, which may be crucial in guiding the selection of immunotherapy strategies for patients with colorectal cancer.

**Conclusion:**

This study, through a comprehensive analysis of colorectal cancer microbiome, immune microenvironment, and drug sensitivity, enhances the current understanding of the multidimensional interactions of colorectal cancer and provides important clinical indications for improving future treatment strategies. The findings offer a new perspective on improving treatment response and long-term prognosis of patients with CRC through the regulation of microbiota or the utilization of biomarkers provided by it.

## Introduction

Colorectal cancer is a prevalent form of cancer worldwide, presenting a significant risk to humans. According to reports from the World Health Organization, colorectal cancer ranks among the top in terms of incidence as well as mortality rates of cancers worldwide (Sung et al., 2021). The etiology of colorectal cancer is still not elucidated completely, but it is known to involve several genetic and environmental factors, including family history, intestinal inflammation, and dietary habits, which considerably increase the risk of its occurrence (Venugopal and Carethers, 2022). Emerging studies suggest that lifestyle factors such as sedentary behavior and obesity also play critical roles in the modulation of this risk, further complicating the interaction between genetics and environmental influences. Recent advancements in high-throughput sequencing technology and extensive research have provided mounting evidence linking the human gut microbiota to the development of colorectal cancer (Qu et al., 2023). This burgeoning field of research has begun to decode the complex dialogues between gut microbial communities and host cellular pathways that may contribute to carcinogenic processes. However, despite significant progress, the specific microbial species and their mechanisms of action in colorectal carcinogenesis remain poorly understood, highlighting the need for more targeted studies in this area.

The gut microbiota refers to the microbiome present in the human or animal intestines, and includes bacteria, fungi, viruses, and other microorganisms (Adak and Khan, 2019). The impact of the human gut microbiota on health and disease states is increasingly being recognized (Illiano et al., 2020). These microorganisms are essential for maintaining the health of the host by engaging in a range of bodily functions like digesting food, absorbing nutrients, and regulating the immune system (Zhao et al., 2019; Zhou et al., 2021). This symbiotic relationship underscores a critical balance, where disruptions can lead to significant health issues, including metabolic and autoimmune diseases. Recent research has shown a strong association between gut bacteria and the onset and progression of different illnesses. Moreover, studies have begun to illustrate how variations in microbiota composition can influence systemic inflammation and immune tolerance, which are pivotal in the context of oncogenesis. Research indicates that particular intestinal bacteria could contribute to the onset and progression of colorectal cancer, potentially influencing the response of patients to cancer treatments like chemotherapy and immunotherapy (Yi et al., 2021; Wong and Yu, 2023). For instance, certain gut bacteria can produce pro-inflammatory factors and trigger chronic inflammatory responses, leading to cancer development (Bishehsari et al., 2020). These responses are often mediated by specific bacterial metabolites that interact with immune cell receptors and signaling pathways involved in inflammation and tumorigenesis. Certain bile acid metabolites or toxins produced by the gut can directly damage the host DNA, increase mutation rates, and promote carcinogenesis. These microbial byproducts could function as cancer-causing agents, impacting the growth and death of cells lining the intestine, altering the biology of intestinal cells (Fang et al., 2021). Research has also shown that gut microbiota dysbiosis can weaken intestinal barrier function, facilitating the entry of bacterial strains or their products through the intestinal wall into the bloodstream, affecting distant cellular populations (Gasaly et al., 2021).

The microbiome in the gut has the ability to influence the behavior of immune cells within the tumor environment, including influencing the roles of macrophages and dendritic cells associated with tumors, consequently interfering with tumor growth and spread (Zhou and Li, 2023). The structure and properties of the tumor microenvironment (TME) have been extensively studied to understand its impact on cancer treatment. The TME plays a vital role in the progression of colorectal cancer, involving a diverse range of immune cells like T cells, B cells, and macrophages. These cells are essential for triggering and maintaining immune responses and suppressing immune activity (Bu et al., 2022). Additionally, the features of TME are considered potential biomarkers for predicting the responses of patients to immunotherapies such as immune checkpoint inhibitors (ICIs). Distinct compositions of gut microbiota have been linked to the response of patients to ICI medications, with variations in gut microbiota potentially impacting patients’ receptiveness to immunotherapy (Zhou et al., 2021).

Although the association between the gut microbiome and colorectal cancer is increasingly becoming evident, comprehensive studies on the categorization of colorectal cancer microbiota and its impact on patient survival predictions are, nonetheless, lacking. A thorough understanding of the classification of microbiota and its relationship with immune infiltration may reveal new methods for the treatment and prognosis assessment, which are crucial for personalized and precision medicine. Therefore, this study aimed to identify microbiota classifications associated with survival prognosis through comprehensive utilization of transcriptomic and microbiome data from patients with colorectal cancer. This study additionally aimed to investigate the relationships among these categorizations and immune features of tumors, and how patients react to chemotherapy and immunotherapy, providing more insight into the influence and function of the intestinal microbiome in colorectal cancer.

## Methods

### Acquisition and processing of transcriptomic and microbiome data

RNA expression profiles and clinical data were obtained from The Cancer Genome Atlas Rectum Adenocarcinoma (TCGA-READ) database. The RNA sequencing data were in Transcripts Per Million (TPM) format, and a log2 transformation was performed for subsequent analysis. Data for the microbiome were obtained from the website https://ftp.microbio.me/pub/cancer_microbiome_analysis/TCGA/. The dataset Kraken-TCGA-Voom-SNM-All-Putative-Contaminants-Removed-Data.csv was chosen, along with the sample details in Metadata-TCGA-Kraken-17625-Samples.csv. After filtering samples from TCGA-READ, a total of 99 samples containing survival information were used for model construction. The data was split into training and validation sets in a 5:5 ratio, with the training set containing 49 samples and the validation set containing 50 samples. The validation set was used to assess the stability and accuracy of the model.

### Acquisition and analysis of scRNA-seq data

We obtained a single-cell dataset containing colorectal cancer samples from the GSE166555 dataset in the Gene Expression Omnibus (GEO) database (Uhlitz et al., 2021). Data were analyzed utilizing the Seurat package in R software (version 4.3.3) (Stuart et al., 2019). When assessing cell quality, we specified that the number of mitochondria should not exceed 10%, and established boundaries for Unique Molecular Identifier counts and gene counts to fall between 200 and 20,000 and 200 and 5,000, respectively. Subsequently, data were normalized, and 2000 highly variable genes were selected. To reduce the impact of the cell cycle, we employed the NormalizeData, FindVariableFeatures, and ScaleData functions in the Seurat package, specifying the vars.to.regress parameter as c(‘S.Score’, ‘G2M.Score’). Batch effects were addressed using the harmony method. The uniform manifold approximation and projection (UMAP), t-distributed stochastic neighbor embedding (t-SNE), and Louvain clustering algorithms to reduce dimensionality and clustering in further analysis. Utilizing the FindAllMarkers function, differential genes were identified across various clusters or cell types based on specified criteria, including p-value<0.05, log2FC>0.25, and expression proportion > 0.1.

### Cell annotation

We utilized a series of cell markers to identify different cell types, and then filtered immune cells for further analysis. The remaining cells were subjected to re-clustering analysis. The mast cells were identified and Sc-Type cell annotation software was employed to annotate the remaining cells. Finally, t-SNE plots and bubble plots of marker genes were generated to visualize the annotation results. The cell markers utilized herein included epithelial cells (EPCAM, KRT18, KRT19, CDH1), natural killer (NK) cells (NCAM1, NKG7, GNLY, KLRD1), fibroblasts (DCN, THY1, COL1A1, COL1A2), T cells (CD3D, CD3E, TRAC, CD3G), endothelial cells (FLT1, CLDN5, RAMP2, PECAM1), myeloid cells (LYZ, MARCO, CD68, FCGR3A), B cells (CD79A, IGHM, IGHG3, IGHA2), and mast cells (MS4A2, KIT, GATA2).

### Identifying microbiome-related tumor subtypes

First, univariate Cox analysis was performed to identify genes linked to patient survival within the microbiome-related gene set (P < 0.05). Afterward, the gene expression matrix was used to conduct consensus clustering on the microbiome data using the ConsensusClusterPlus package (Wilkerson and Hayes, 2010). We selected the k-means method and utilized Euclidean distance as the similarity measure for the clustering algorithm. Then, 100 bootstrap samplings were conducted, each comprising 80% of the samples. The clustering numbers ranging from 2 to 6 were tested and the optimal classification was determined using the Proportion of Ambiguous Clustering (PAC) and consistency matrix.

### Construction and validation of the prognostic model

The limma package was utilized to calculate the differences in microbiome composition between varying tumor subtypes (Ritchie et al., 2015). Next, we employed univariate Cox analysis to identify prognosis-linked microbiota (P < 0.05). To decrease the amount of microbiota, we utilized the Glmnet package to conduct the Least Absolute Shrinkage and Selection Operator (LASSO)-Cox regression analysis (Engebretsen and Bohlin, 2019). We further reduced the number of microbiota using Stepwise Cox regression analysis (StepCox). Ultimately, we derived a formula to determine the risk score, RiskScore = β1 * exp1 + β2 * exp2 +… + βi * expi. In this context, β symbolizes the microbiota coefficient, and ‘exp’ signifies the microbiota level. Using this equation, the risk scores in the TCGA training set was calculated. Following that, we carried out receiver operating characteristic (ROC) assessment using the timeROC package (Blanche et al., 2013), illustrating ROC curves for 1, 2, and 3 years, and executing survival examination with the survminer package to establish the threshold value (Kassambara et al., 2021). Finally, the stability of the prognostic model was validated using the TCGA test set and the entire TCGA dataset.

### Gene set enrichment analysis and functional annotation

The clusterProfiler package (Wu et al., 2021) was used to perform GSEA on the upregulated genes of various tumor subtypes to evaluate their functional characteristics. We used gene sets from the Kyoto Encyclopedia of Genes and Genomes (KEGG) database as enrichment signatures, considering a functional enrichment significance when the adjusted p-value after the Benjamini-Hochberg correction was <0.05. We utilized the enrichplot package for visualization and the Single Sample Gene Set Enrichment Analysis (ssGSEA) algorithm from the Immuno-Oncology Biological Research package to compute functional scores for individual samples. We performed differential analysis between tumor subtypes/risk groups using the Hallmark gene set. For this assessment, we employed the Wilcoxon test, deeming a p-value < 0.05 to be statistically significant.

### Tumor immune infiltration analysis

Metrics related to immune infiltration in the The Cancer Genome Atlas (TCGA)-READ dataset, including StromalScore, ImmuneScore, ESTIMATEScore, and TumorPurity, were computed using the ESTIMATE algorithm. The immune cell composition in TCGA-READ was assessed using Cell-type Identification By Estimating Relative Subsets Of RNA Transcripts (CIBERSORTx). Using single-cell data on immune cell composition as a reference, we predicted the immune composition of bulk samples. Comparisons between different groups were conducted using the Kruskal-Wallis test. We utilized the pheatmap package to visualize Estimation of STromal and Immune cells in MAlignant Tumor tissues using Expression data (ESTIMATE) score distribution and immune cells across tumor subtypes, as well as the distribution of ssGSEA scores for signatures such as Hallmark between risk groups (Kolde, 2019).

### Prediction of the response to immunotherapy/chemotherapy

The oncoPredict package in R was used to determine the IC50 of standard chemotherapy medications for evaluating the effectiveness of chemotherapy. The T-cell-inflamed gene expression profile score was utilized to forecast the reaction to immunotherapy in various tumor subtypes and risk groups. Next, the cytolytic activity (CYT) score and type 1 T helper/interferon gamma (Th1/IFNγ) gene signature score was computed utilizing the single-sample GSEA (ssGSEA) algorithm. Additionally, the Tumor Immune Dysfunction and Exclusion (TIDE) web analysis tool from http://tide.dfci.harvard.edu/ was utilized to forecast the immune reaction and ratings in the TCGA dataset. Immune checkpoint genes were used to analyze differentially using the Kruskal-Wallis test, with a padj<0.05 deemed as statistically significant. Subsequently, a correlation analysis was conducted between immune checkpoint genes and risk scores upon utilizing the ggcorrplot package (Kassambara and Patil, 2023).

Somatic Nucleotide Variant (SNV) analysis.

The TCGA database provided us with data on SNV mutations, and then the maftools package (Mayakonda et al., 2018) was used to analyze and determine tumor mutation burden (TMB), mutant-allele tumor heterogeneity (MATH), and homologous recombination defects (HRD) for every sample. Furthermore, a comparative analysis among tumor subtypes/risk groups was performed utilizing the Wilcoxon test; a p-value<0.05 was deemed as statistically significant. Additionally, a correlation analysis was conducted between risk scores and immune cells, as well as the Hallmark signature, utilizing the ggcorrplot tool.

## Results

### Consensus clustering analysis of microbiota-associated tumor subtypes

**Figure 1:**
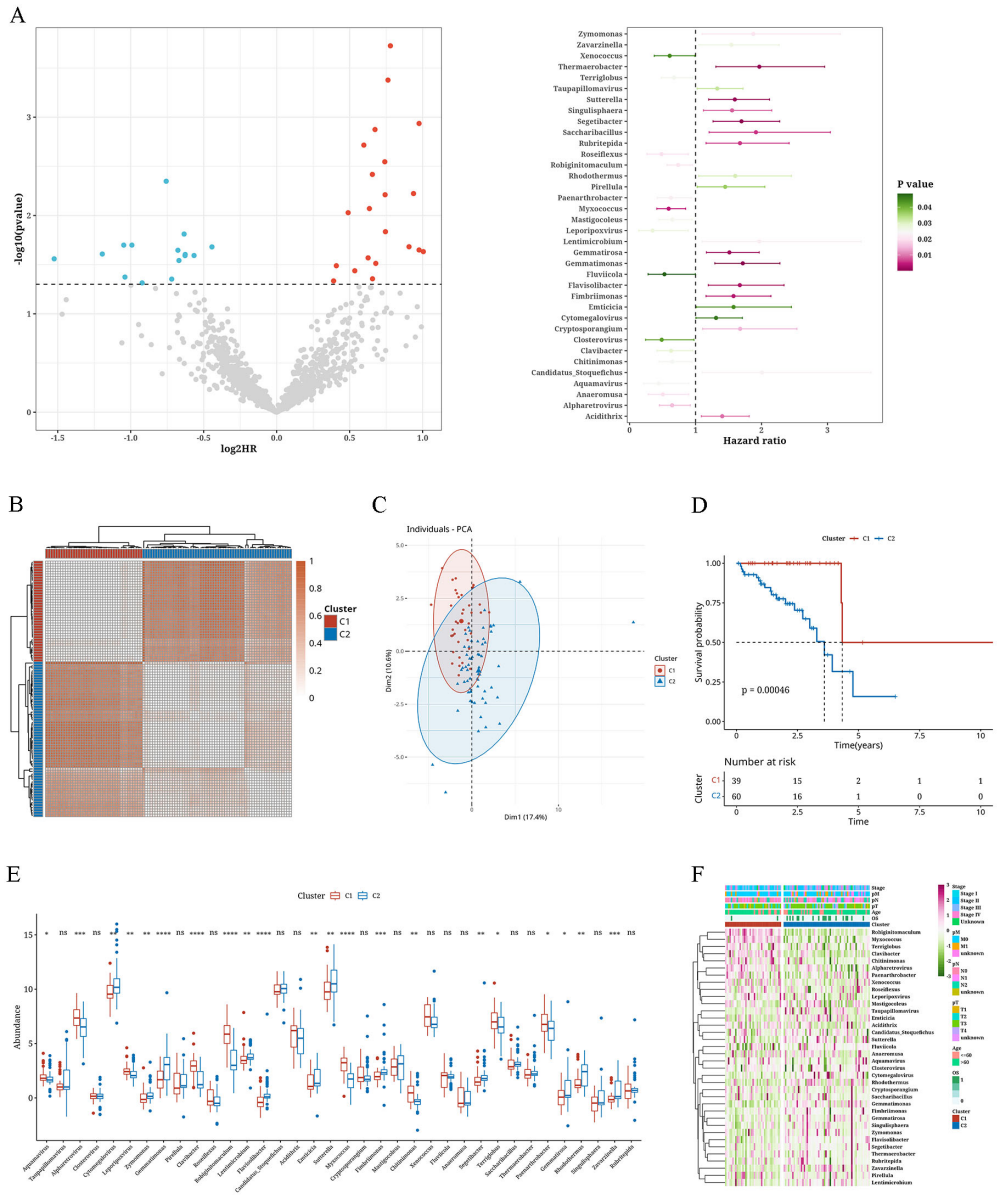
Consensus clustering analysis of microbiota-associated tumor subtypes. **(A)** Volcano plot and error bar graph representing survival-associated microbiota. **(B)** Consistency clustering plot of microbial abundance matrix. **(C)** Principal Component Analysis plot with K=2. **(D)** Survival analysis curves of two clusters. **(E)** Box plot showing differences in abundance of microbiota between two clusters. **(F)** Heatmap depicting the abundance of microbiota between two clusters. *: p<0.05; **: p<0.01; ***: p<0.001; ****:p<0.0001.

Microbial genera associated with survival were identified through univariate Cox analysis of the microbiome data (P < 0.05) (**Figure 1A**). Subsequently, consensus clustering on the TCGA data was performed utilizing the abundance matrix of genera and the ConsensusClusterPlus package. Through PAC analysis, we found that the best grouping effect occurred with a value of k=2 (**Figure 1B**). Principal component analysis revealed the distribution of samples from the two subtypes (**Figure 1C**). Survival analysis results showed poorer prognosis of subtype C2 (p=0.00046) (**Figure 1D**). Furthermore, significant differences were observed between the two subtypes in terms of the abundance expression of most genera (**Figure 1E**). The abundance heatmap of genera revealed that *Robiginitomaculum*, *Myxococcus*, *Terriglobus*, *Clavibacter*, *Chitinimonas*, *Alpharetrovirus*, *Paenarthrobacter*, and *Xenococcus* had higher abundance in C1, while the remaining genera (Cytomegalovirus, Zymomonas, Lentimicrobium, Flavihumibacter, Emticicia, Sutterella, Fimbriimonas, Segetibacter, Gemmatirosa and Zavarzinella) had higher abundance in C2 (**Figure 1F**).

### Variations in clinical markers and single nucleotide variations across tumor subtypes

**Figure 2:**
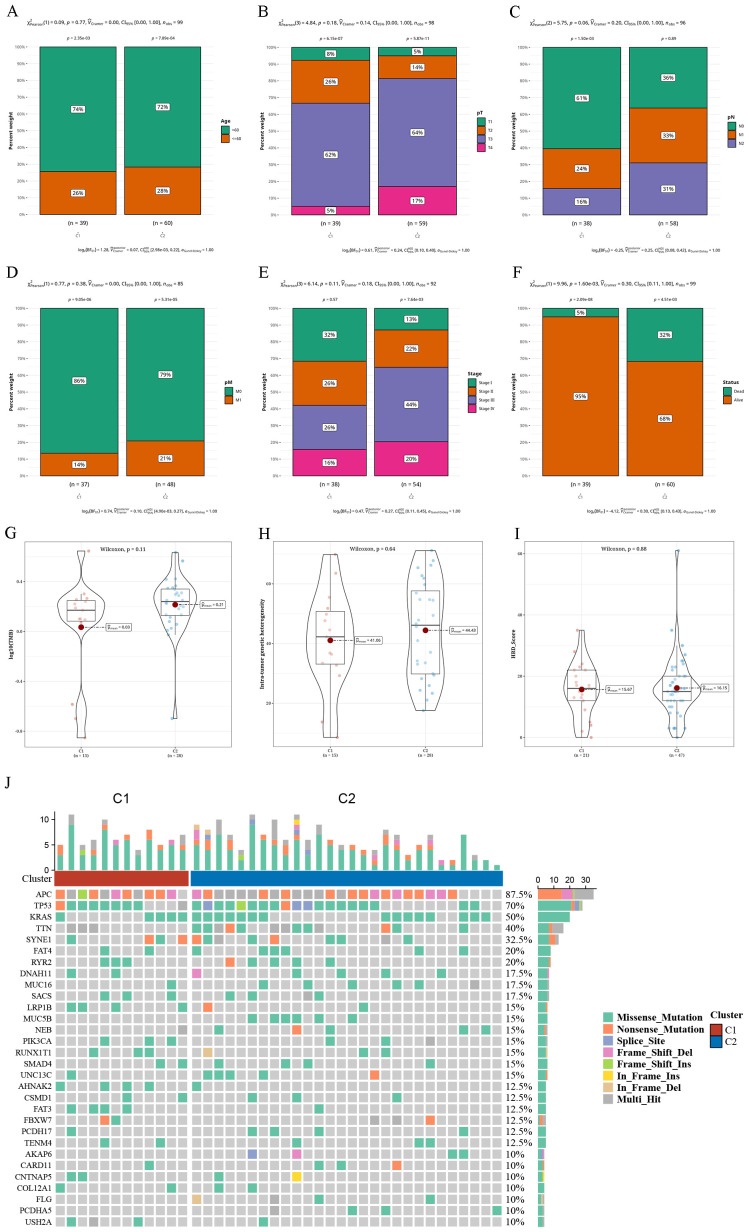
Differential clinical indicators and somatic nucleotide variants mutations in tumor subtypes **(A–F)** Bar charts illustrating the proportion of various clinical indicators (age, pT, pN, pM, Tumor Stage, survival status) in two clusters. **(G–I)** Violin plots displaying variances in tumor mutation burden (TMB), mutant-allele tumor heterogeneity (MATH), and homologous recombination defects (HRD) between two clusters. **(J)** Waterfall plot depicting the top 30 gene mutations in two clusters.

We combined the subtype outcomes with various clinical factors, including age, tumor stage, survival status, and of T, N, and M stages. Chi-square tests revealed that the survival status distribution differed significantly between C1 and C2 through (P < 0.05), and other factors showed near significance (**Figures 2A–F**). The SNV mutations in these two subtypes were calculated in terms of tumor mutation burden (TMB), mutant-allele tumor heterogeneity (MATH), and homologous recombination defects (HRD) values. No notable variances were observed between C1 and C2 in (**Figures 2G–I**). The waterfall chart displays the mutation status of the most frequently mutated top 30 genes in both subtypes. We observed that the genes Adenomatous Polyposis Coli (*APC)* (87.5% mutation rate), *TP53* (70% mutation rate), and *KRAS* (50% mutation rate) exhibited relatively high mutation rates (**Figure 2J**).

### Functional differences in tumor subtypes

**Figure 3:**
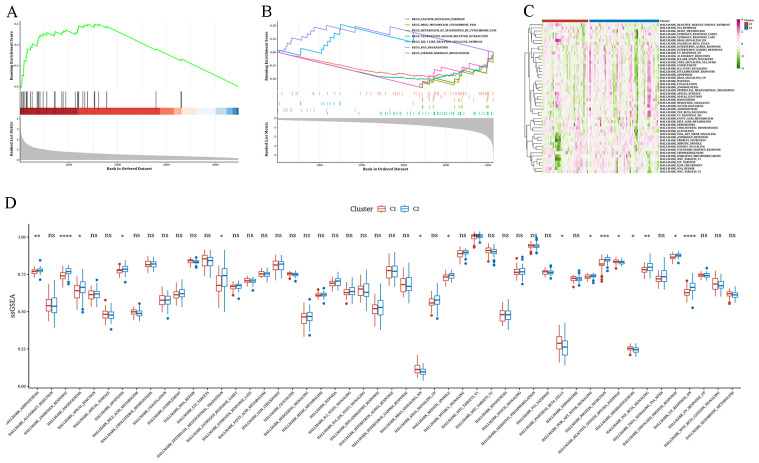
The results of functional differential analysis of tumor subtypes **(A)** Gene Set Enrichment Analysis (GSEA) results of upregulated genes in cluster 1 (C1). **(B)** GSEA results of upregulated genes in cluster 2 (C2). **(C)** Heatmap displaying single-sample GSEA (ssGSEA) scores for samples categorized into two clusters using the Hallmark gene set. **(D)** Boxplot illustrating the variation in ssGSEA scores between samples in two clusters using the Hallmark gene set. *: p<0.05; **: p<0.01; ***: p<0.001; ****: p<0.0001.

After comparing gene expression differences between the two subtypes, we utilized the marker genes identified for GSEA to assess their functional importance through KEGG analysis. According to this enrichment analysis, subtype C1 was enriched in the KEGG_NEUROACTIVE_LIGAND_RECEPTOR_INTERACTION pathway, whereas subtype C2 showed enrichment in the KEGG_RNA_DEGRADATION, KEGG_METABOLISM_OF_XENOBIOTICS_BY_CYTOCHROME_P450, and KEGG_RIG_I_LIKE_RECEPTOR_SIGNALING_PATHWAY pathways (**Figures 3A, B**). Further evaluation of key pathways revealed that ADIPOGENESIS, ANDROGEN_RESPONSE, ANGIOGENESIS, APOPTOSIS, and EMT were more prevalent in subtype C2, whereas REACTIVE_OXYGEN_SPECIES_PATHWAY, PANCREAS_BETA_CELLS, and KRAS_SIGNALING_DN were more prevalent in subtype C1 (**Figures 3C, D**).

### Immune infiltration differences among tumor subtypes

**Figure 4:**
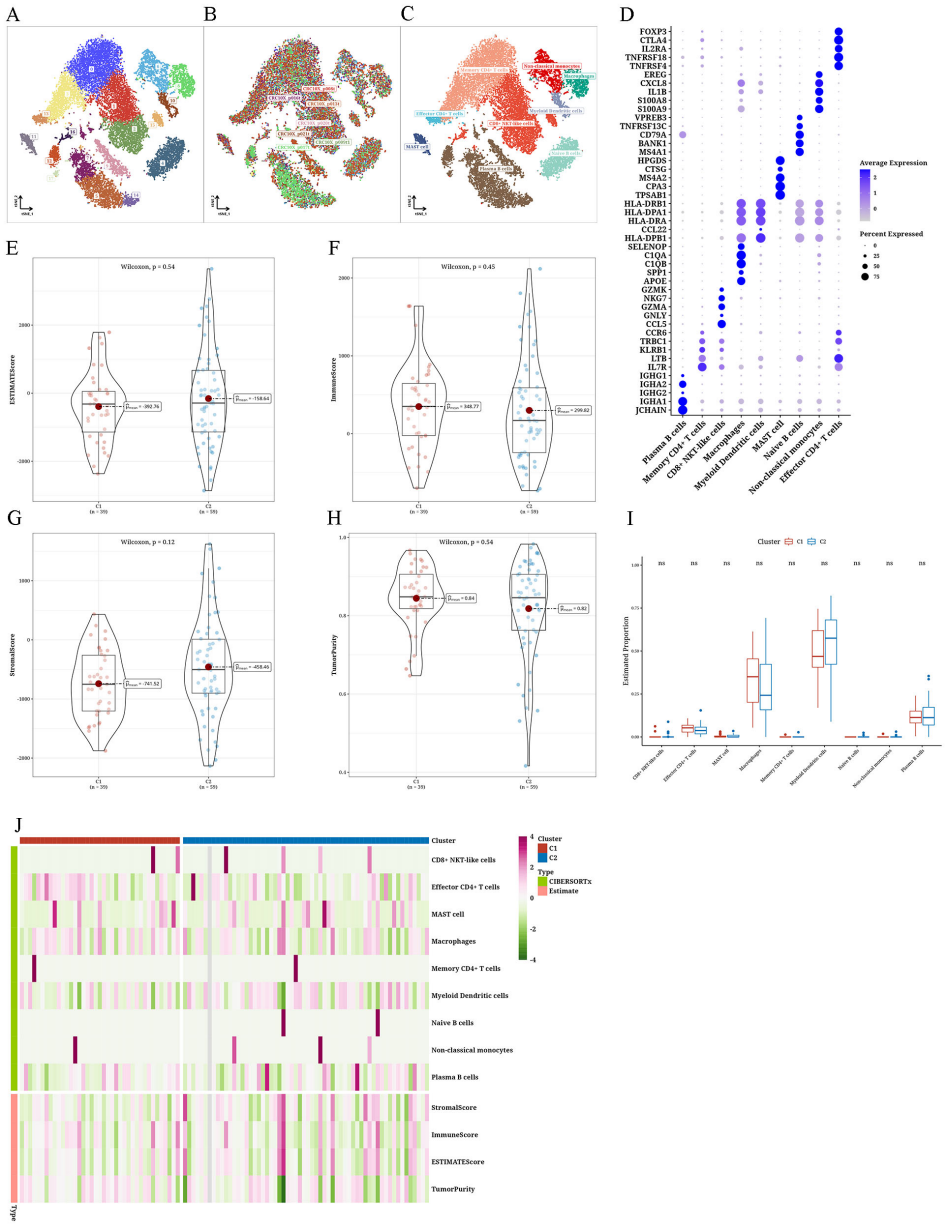
Differential immune infiltration results of tumor subtypes **(A–C)** The t-distributed stochastic neighbor embedding plots of single-cell data from The Cancer Genome Atlas Rectum Adenocarcinoma (TCGA-READ). **(D)** Bubble plot showing marker annotations for various cell types. **(E)** Violin plot depicting the differential Estimation of STromal and Immune cells in MAlignant Tumor tissues using Expression data (ESTIMATE) score between two clusters. Violin plot illustrating the differential **(F)** ImmuneScore between two clusters, **(G)** StromalScore between two clusters, and **(H)** TumorPurity between two clusters. **(I)** Boxplot showing the differential CIBERSORTx scores between two clusters. **(J)** Heatmap presenting the differential analysis results of ESTIMATE and CIBERSORTx scores between two clusters. ns: No statistical significance.

analysis of single-cell data of READ, particularly focusing on isolating immune cells, resulted in a total of 18 clusters. Based on the distribution of cell sources, immune cells were derived primarily from tumor samples. Employing distinct markers and the Sc-Type program, we could categorize immune cells and acquire cell annotation outcomes for every cluster. Subsequently, bubble expression plots of markers for various cell types were displayed (**Figures 4A–D**). The ESTIMATE algorithm produced four indices that showed no significant differences between the two subtypes. However, ImmuneScore and Tumor-Purity were higher in C1, while StromalScore and ESTIMATEScore were higher in C2 (**Figures 4E–H**). Employing the CIBERSORTx algorithm and leveraging single-cell data as a reference to predict bulk data, we found higher levels of macrophages in C1, whereas myeloid dendritic cells (mDCs) were more abundant in C2 (**Figures 4I, J**).

### Examining anticipated reaction to immunotherapy and chemotherapy in different types of tumors

**Figure 5:**
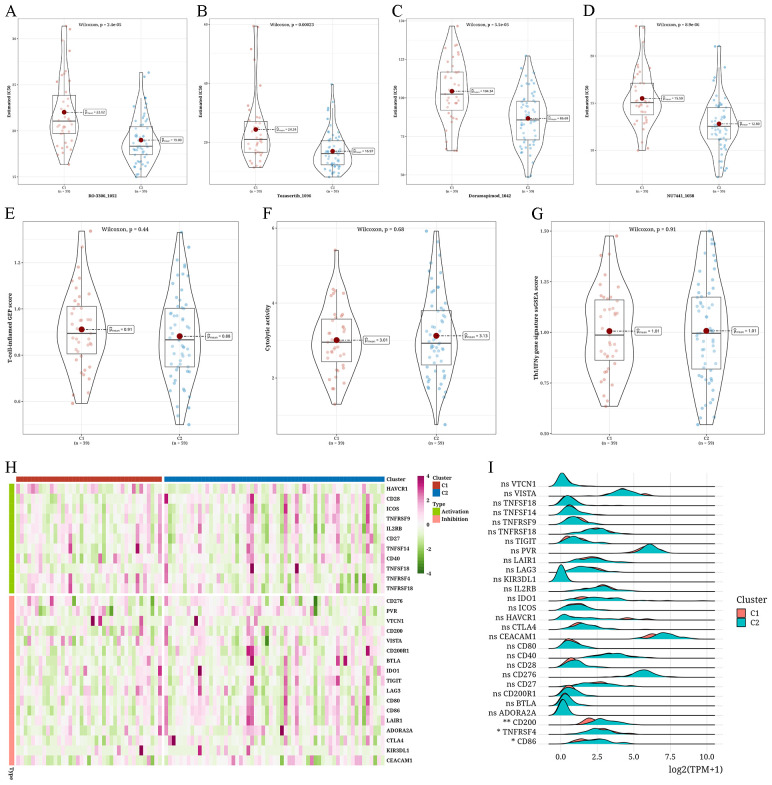
Assessment of anticipated reaction to immunotherapy and chemotherapy in different types of tumors **(A–D)** Violin plots depicting the differential IC50 values of RO-3306_1052, Tozasertib_1096, Doramapimod_1042, and NU7441_1038 between two clusters (C1 and C2). **(E–G)** Violin plots showing the differences in T-cell-inflamed gene expression profile (GEP) score, cytolytic score (CYT) score, and type 1 T helper/interferon-gamma (Th1/IFNγ) gene signature between two clusters. **(H)** Heatmap showing the expression levels of 28 immune checkpoint genes across different subtypes. **(I)** A Ridge plot illustrating the levels of expression for 28 immune checkpoint genes among various subtypes (*p < 0.05; **p < 0.01; ns, not significant).

For every subtype, the IC50 values of traditional chemotherapy medications were determined. In C2, RO-3306_1052, Tozasertib_1096, Doramapimod_1042, and NU7441_1038 exhibited higher sensitivity levels (**Figures 5A–D**). Assessment of the reaction to immunotherapy through the T-cell-inflamed Gene Expression Profile (GEP) score, CYT score, and Th1/IFNγ gene signature showed that C1 had a greater T-cell-inflamed gene expression profile score than C2, whereas C2 had a higher cytolytic activity score compared to C1. Both subtypes exhibited comparable levels of Th1/IFNγ gene signature scores (**Figures 5E–G**). We then examined the levels of 28 immune checkpoint genes in every subtype and observed significant variations in the levels of *CD200*, *TNFRSF4*, and *CD86* immune checkpoint genes between the two subtypes, and all three were more abundant in C2 (**Figures 5H, I**).

### Construction and validation of risk score models

**Figure 6:**
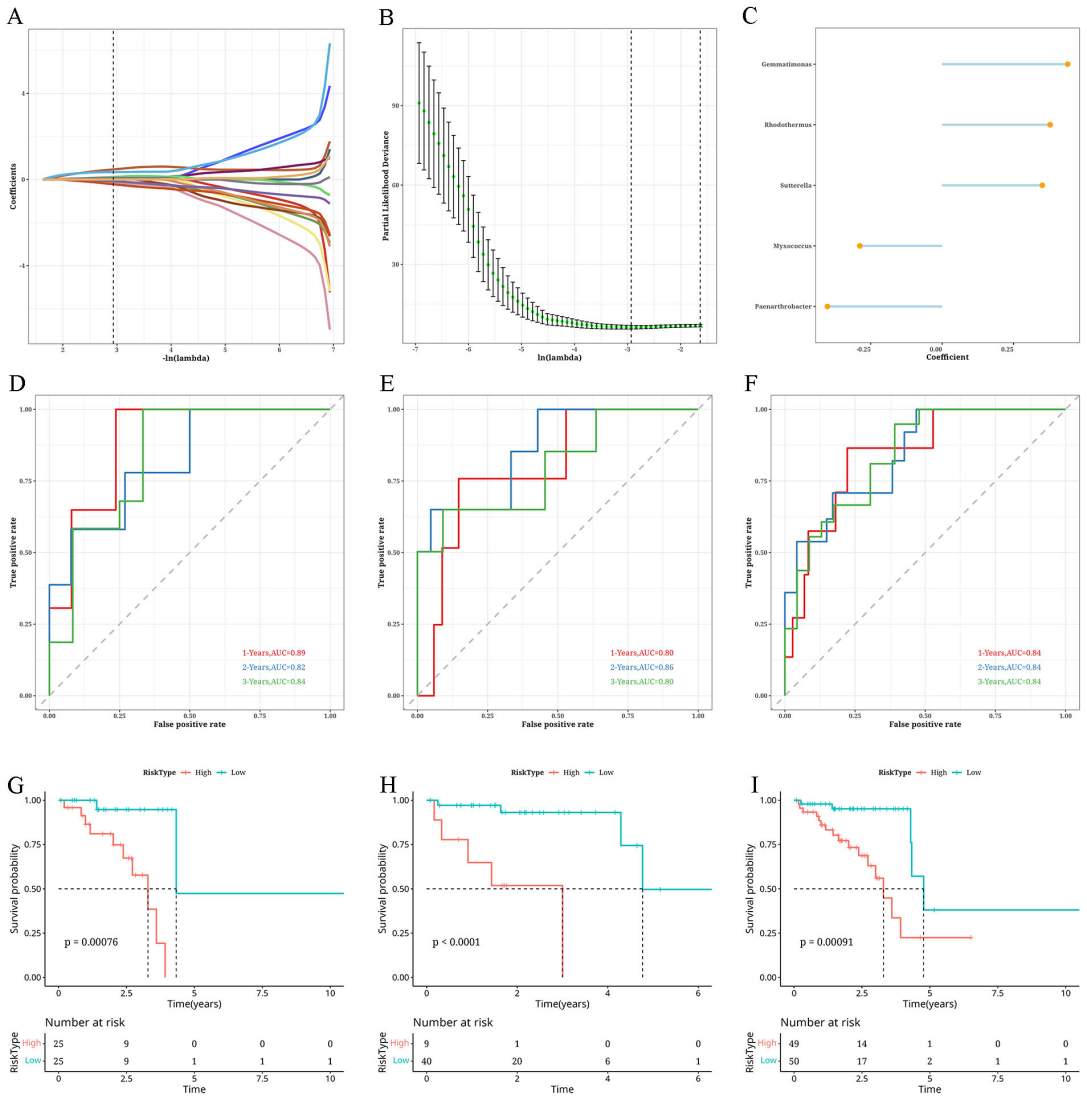
Analysis of building and validation of risk score models **(A)** A Curve plot showing the coefficient of prognostic genera with respect to lambda values. **(B)** An error bar graph showing the partial likelihood deviance for the LassoCox model at different lambda values. **(C)** A bubble plot showing the coefficient values of genera in the risk score model. **(D)** Survival analysis findings from the TCGA validation dataset. **(E)** Survival analysis findings from the TCGA validation dataset. **(F)** Findings from the survival analysis of the complete TCGA database. **(G)** The timeROC analysis results for the 1, 2, and 3-year intervals in the TCGA testing set. **(H)** The timeROC analysis results for the 1, 2, and 3-year intervals in the TCGA testing set. **(I)** The timeROC analysis results for the 1, 2, and 3-year intervals in the entire TCGA dataset.

Based on differential analysis between the two subtypes (|log2FC|>0.5, P<0.05), we identified 133 distinct genera. Univariate Cox analysis identified 16 genera exhibiting significant associations with survival (P<0.05), with 9 genera linked to risk and 7 linked to protection. Following this, a model was created using the LASSO-Cox regression technique, leading to a decrease in the number of genera. With the increase in the λ coefficient, the number of genera approached zero (**Figure 6A**). Ten-fold cross-validation yielded confidence intervals for each λ value (**Figure 6B**). Ultimately, nine prognostic-related genera could be identified. StepCox was used to enhance the genera selection, leading to the following equation for RiskScore calculation: RiskScore = 0.44*Gemmatimonas+0.378*Rhodothermus+0.351*Sutterella-0.288*Myxococcus-0.402*Paenarthrobacter (**Figure 6C**). Applying this formula, we determined the risk levels of the TCGA training set, TCGA test set, and the complete TCGA dataset, uncovering a worse outlook for the high-risk category. Furthermore, the estimated Area Under the Curve (AUC) values for 1, 2, and 3 years, were all greater than 0.8 for every dataset (**Figures 6D–I**).

### Variations in clinical markers within different risk groups

**Figure 7:**
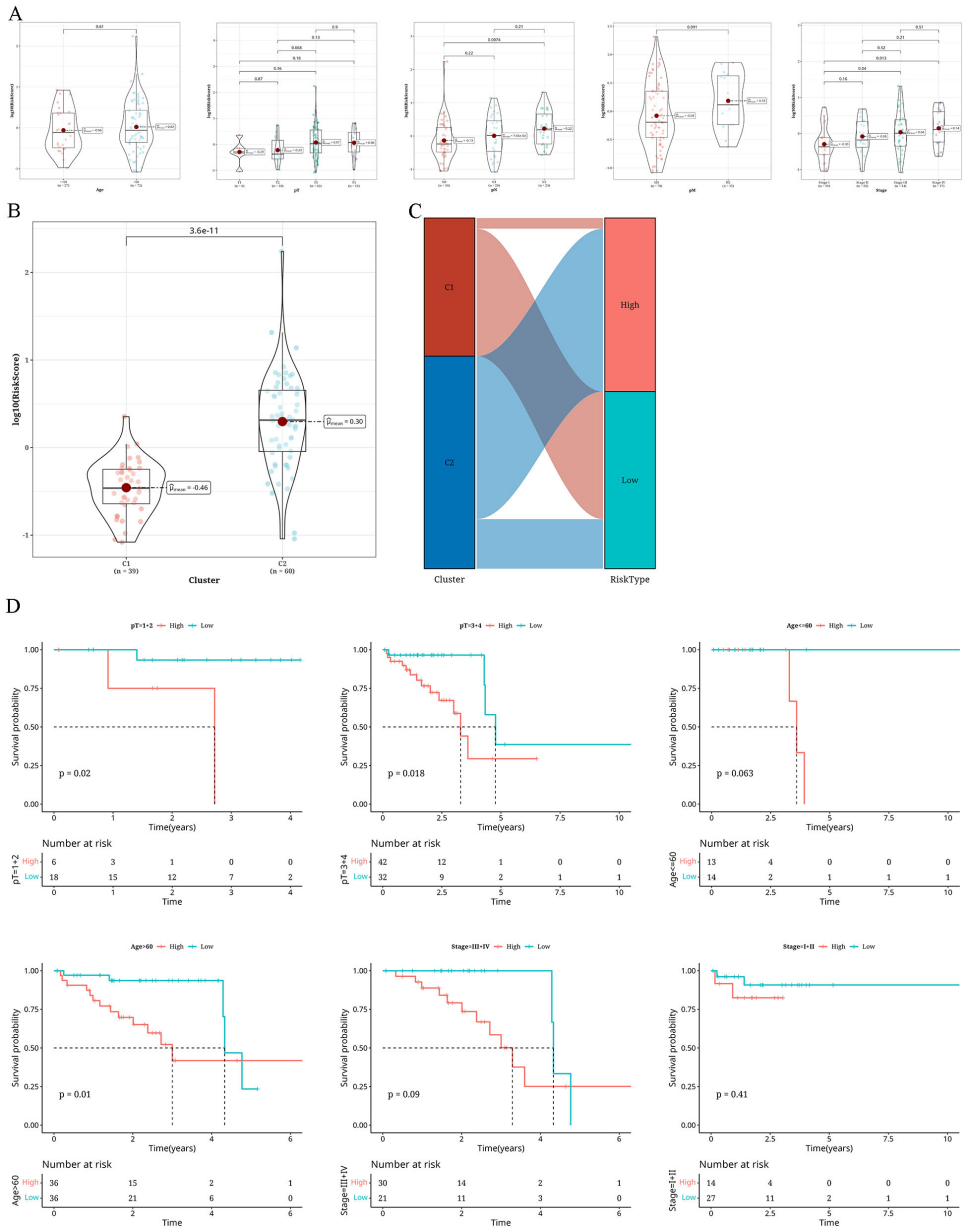
Findings from the examination of distinct clinical markers in various risk categories **(A)** A Violin plot showing the variations in risk levels of different clinical markers (age, pT, pN, pM, Tumor_Stage) within The Cancer Genome Atlas database cohort. **(B)** A violin plot illustrating the variations in risk levels across two distinct clusters (C1 and C2). **(C)** A sankey diagram showing the compositional differences between two clusters and risk groups. **(D)** Survival analysis results classified according to pT, age, and Tumor_Stage.

A combined analysis of the risk groups with various clinical indicators revealed that with the increase in age, T stage, N stage, M stage, pathological grading, and tumor staging, the risk value also increased (**Figure 7A**). In particular, the risk assessment for C2 was notably greater compared to C1 (**Figure 7B**). **Figure 7C** presents a Sankey diagram for the allocation of tumor subtypes and risk categories. Subsequently, for survival analysis, we segregated samples based on pathological T stage, age, and tumor stage in three groups. **Figure 7D** shows a worse outcome of individuals in the high-risk category.

### Differences in immune infiltration among the risk groups

**Figure 8:**
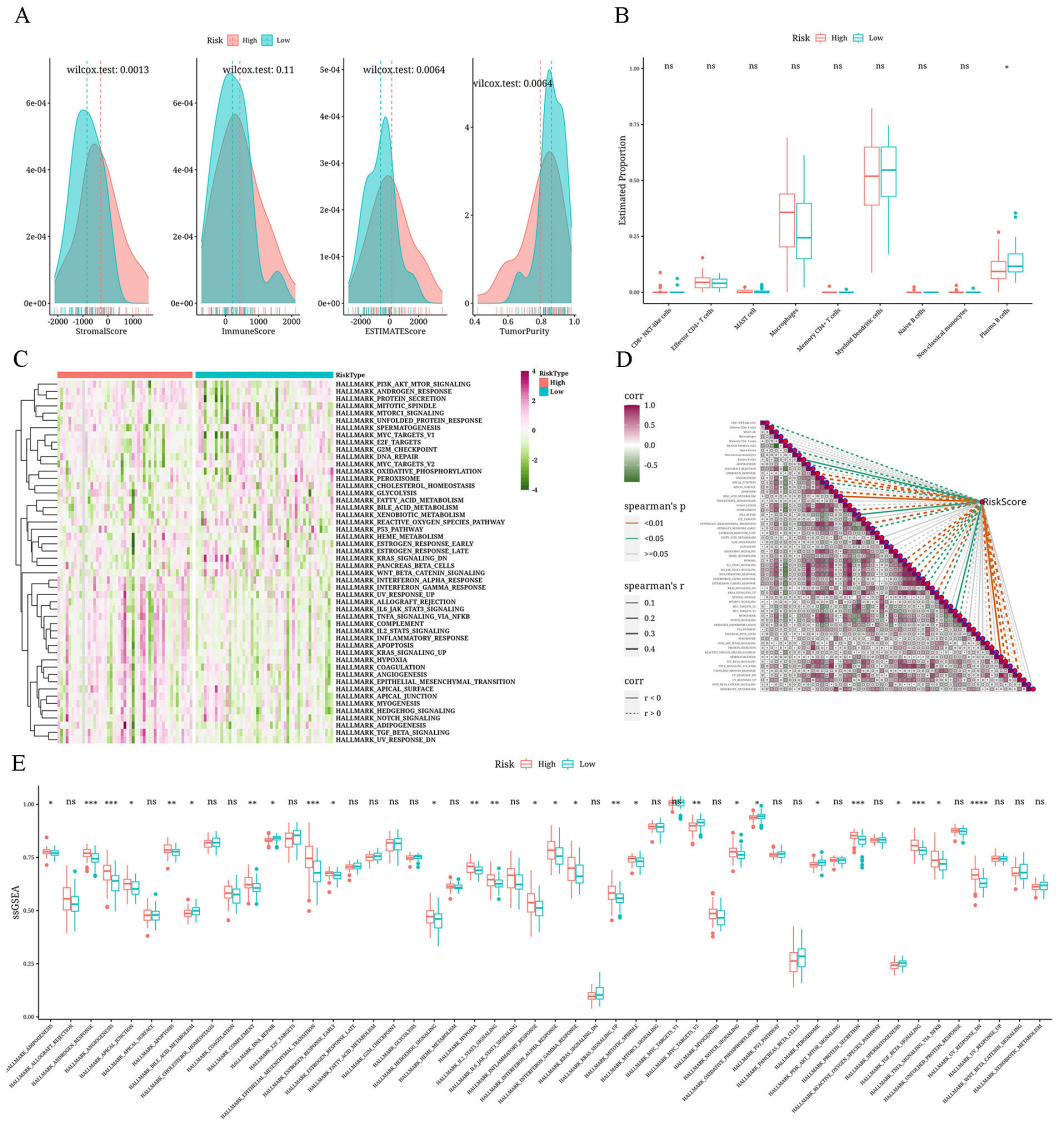
Analysis results of differential immune microenvironment in risk groups **(A)** Density distribution plots depicting StromalScore, ImmuneScore, ESTIMATEScore, and TumorPurity across different risk groups. **(B)** Boxplot showing the differential CIBERSORTx scores between risk groups. **(C)** Heatmap displaying single-sample Gene Set Enrichment Analysis (ssGSEA) scores using the Hallmark gene set for different risk groups. **(D)** Correlation heatmap illustrating the relationship between risk values, immune cell composition, and Hallmark gene set (ssGSEA scores) (positive correlation is shown as a dashed line and negative correlation, as a solid line). **(E)** A boxplot displaying the differences among different risk groups in terms of ssGSEA scores for the Hallmark gene set. *: p<0.05; **: p<0.01; ***: p<0.001; ****: p<0.0001.

The three matrices computed through the ESTIMATE algorithm exhibited notable variances among the risk categories (P<0.05), and no significant variance was detected in the ImmuneScore. In particular, the values for StromalScore, ImmuneScore, and ESTIMATEScore were elevated in the high-risk category, whereas the Tumor-Purity score was increased in the low-risk category (**Figure 8A**). Applying the CIBERSORTx algorithm to predict bulk information employing single-cell data as a guide, we discovered a greater prevalence of macrophages in the high-risk category, whereas mDCs and plasma B cells were more prevalent in the low-risk category (**Figure 8B**). The pathway enrichment analysis revealed enrichment of pathways like MYC_TARGETS_V2 and BILE_ACID_METABOLISM in the low-risk group, whereas pathways like ANDROGEN_RESPONSE and ANGIOGENESIS were enriched in the high-risk group (**Figures 8C, E**). Following this, the Spearman correlation analysis showed a negative correlation of the risk-score with plasma B cells, BILE_ACID_METABOLISM, and other factors, concurrently showing a positive correlation with CD8+ NKT-like cells, naive B cells, ADIPOGENESIS, and other factors. As shown in **Figure 8D**, a positive correlation is indicated by the dashed line, while a negative correlation is represented by the solid line.

### Evaluation of anticipated reaction to immunotherapy/chemotherapy within the risk groups

**Figure 9:**
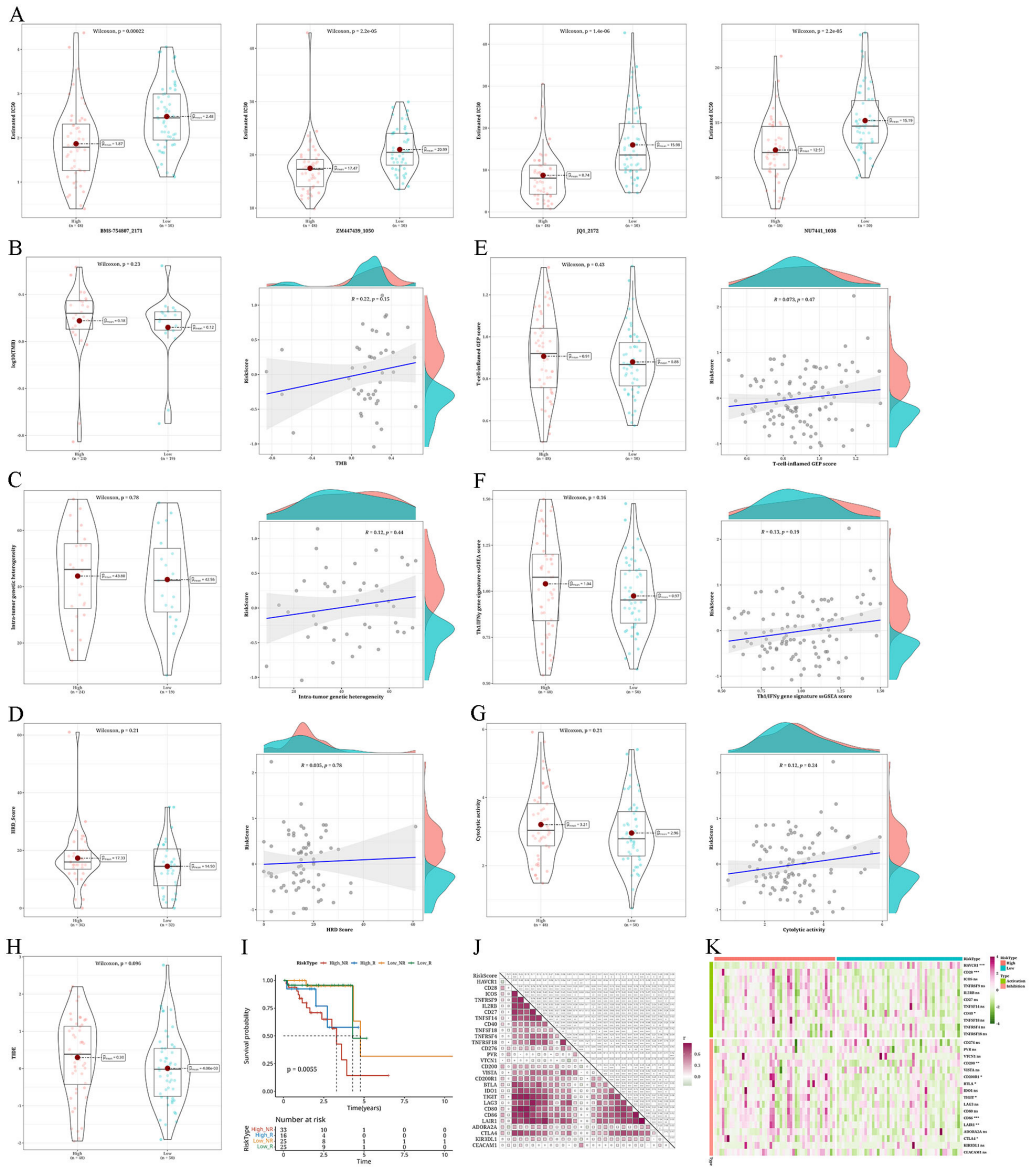
Analysis of anticipated reaction to immunotherapy/chemotherapy and variations in somatic nucleotide variants (SNV) mutations among different risk categories **(A)** Violin plots depicting the differential IC50 of BMS-754807_2171, ZM447439_1050, JQ1_2172, and NU7441_1038 between risk groups. **(B–D)** Violin plots showing the differences in tumor mutation burden (TMB), mutant-allele tumor heterogeneity (MATH), and homologous recombination defects (HRD) between risk groups, along with corresponding correlation scatter plots. **(E–G)** Violin plots demonstrating variations in T-cell–inflamed gene expression profile (GEP) score, type 1 T helper/interferon gamma (Th1/IFNγ) gene signature, and cytolytic score (CYT) score among different risk groups, accompanied by correlation scatter plots. **(H)** Violin plot displaying the differences in TIDE values between high- and low-risk groups. **(I)** Survival analysis results of risk groups categorized as Response and NonResponse predicted by combining risk groups with TIDE analysis. **(J)** A heatmap displaying the association between risk values and the expression levels of immune checkpoint genes. **(K)** Heatmap illustrating the expression levels of immune checkpoint genes in patients categorized by risk groups (*p < 0.05; **p < 0.01; ***p < 0.001; ****p < 0.0001; ns, not significant).

In the high-risk group, enhanced sensitivity was observed for BMS-754807_2171, ZM447439_1050, JQ1_2172, and NU7441_1038 (**Figure 9A**). The mutation analysis exhibited elevated levels of TMB, MATH, and HRD scores in the high-risk group in comparison to the low-risk group. Furthermore, all three metrics had a positive association with the risk factor, although this relationship was not statistically significant, as shown in **Figures 9B–D**. Investigation of immunotherapy revealed elevated levels of T-cell–inflamed GEP score, Th1/IFNγ gene signature, and CYT score in the high-risk group compared to the low-risk group. These three factors were all linked to the risk value, although the association was not statistically significant (**Figures 9E–G**). Using TIDE online analysis, we discovered significantly higher TIDE value in the high-risk group compared to the low-risk group, with results nearing statistical significance (**Figure 9H**). Survival analysis showed a worse prognosis for the high-risk non-responding group (**Figure 9I**). During the examination of immune checkpoints, the level of risk showed a strong association with the majority of immune checkpoints, and the high-risk group had a notably higher presence of immune checkpoints (**Figures 9J, K**).

## Discussion

Colorectal cancer is a globally prevalent type of cancer; it is the third most frequently detected cancer, according to the World Cancer Research Fund. Traditional treatment methods for colorectal cancer primarily include surgery, radiotherapy, and chemotherapy. Personalized treatment options are also now available for certain patients with the development of targeted therapies and immunotherapy (Benson et al., 2022). However, despite the increasing diversity of treatment modalities, the recurrence and mortality rates of colorectal cancer remain high. A strong link has been shown between the intestinal flora of individuals with colorectal cancer and the onset, progression, response to treatment, and outlook of the disease, suggesting a significant impact of the intestinal flora on the treatment of colorectal cancer.

By combining transcriptomic and microbiota data in individuals with colorectal cancer, we could discover two unique tumor subcategories (C1 and C2) linked to varying microbiota compositions that significantly influence patient survival predictions. Individuals classified as the C1 subtype exhibited a more favorable outlook. Individuals with the C2 subtype showed elevated abundance of various genera of gut bacteria, which is potentially linked to tumor development (Lee et al., 2018). In line with earlier research, this discovery implies a strong association between intestinal flora and tumor development, suggesting that gut bacteria could contribute to the occurrence of inflammatory conditions that support tumor growth, influence immune system avoidance, and aid in tumor spread and growth (Dzutsev et al., 2015; Fu et al., 2024). Clinical parameters too, showed differences in survival status between C1 and C2. Nonetheless, the lack of significance in the variances of TMB, MATH, and HRD values between the two subtypes indicates the presence of alternative mechanisms contributing to the differences in subtypes. Additionally, the *APC* gene was identified with a high mutation rate. APC, a key gene involved in tumor suppression, is essential in regulating intestinal mucosal epithelial cells by regulating cell proliferation, differentiation, apoptosis, and cell cycle. Mutation or loss of the *APC* gene can lead to dysregulated cell cycle, thereby promoting the occurrence of colorectal tumors (Goss and Groden, 2000; Malki et al., 2020).

Tumors with a poor prognosis often have a higher mutational burden, meaning they present more neoantigens on their surface. These neoantigens make the tumor cells more recognizable to the immune system. ICIs work by blocking immune checkpoint molecules (like PD-1 and CTLA-4), which removes the brakes on T cells, enabling them to attack these highly mutated tumors more effectively. Poor prognosis tumors may have developed mechanisms to escape immune surveillance, often involving overexpression of immune checkpoint molecules. ICIs can reverse this immune escape by blocking these checkpoints, leading to reactivation of the immune response against the tumor. The heterogeneity within tumor cells might mean that certain subgroups within a poor prognosis tumor are particularly sensitive to ICIs. Patients with a poor prognosis might have a higher proportion of these sensitive subgroups, resulting in a better response to ICIs. Poor prognosis tumors that do not respond well to conventional therapies (like chemotherapy or radiation) might still undergo immunogenic cell death. This form of cell death releases tumor antigens, which can further activate the immune system, thereby enhancing the effectiveness of ICIs. Studies have shown that combining ICIs with other treatments (such as chemotherapy, radiation, or targeted therapies) can lead to better outcomes, particularly in patients with poor prognosis. These combination therapies might improve the tumor microenvironment or expose more tumor antigens, making ICIs more effective.

Interestingly, by combining microbiota and single-cell data, we uncovered unique immune cell infiltration patterns displayed by tumors categorized into various gut microbiota subgroups. The presence of these patterns might have a direct impact on the advancement of the illness and the outlook for the patient. Our examination showed that while there was no notable variation in immune scores among the two subtypes, the C1 subtype potentially harbored a greater number of macrophages, thus culminating in an enhanced anti-cancer immune reaction; whereas, the C2 subtype showed a higher proportion of Dendritic Cells (DCs), which may suppress anti-tumor responses, thus explaining its association with poorer prognosis.

Response prediction to chemotherapy and immunotherapy shows a close association between drug sensitivity and treatment response in different tumor subtypes. For example, medications like RO-3306, Tozasertib, Doramapimod, and NU7441 have been thoroughly studied and shown to inhibit cancer cells, control cellular stress responses, trigger cancer cell death, and consequently slow down tumor progression (Moon et al., 2015; Matsumoto, 2022; Yang et al., 2022; Huang et al., 2023). The C2 subtype appears to be more sensitive to the above-mentioned chemotherapy drugs, suggesting better treatment outcomes for patients with this subtype upon treatment with these drugs. Additionally, immune checkpoint molecules CD200, TNFRSF4, and CD86 are all enriched in the C2 subtype, while the C1 subtype exhibits enrichment of T-cell inflamed gene expression patterns. These results indicate that patients with the C2 subtype respond better to immunotherapy.

Poor prognosis was confirmed by the AUC values, validating the effectiveness of the model. Combined with clinical parameters, patients with higher-risk clinical-pathological parameters tended to have higher scores. Additionally, the risk scores were higher in the C2 group, consistent with its poorer prognosis. Additionally, within the high-risk category, the levels of StromalScore, ImmuneScore, and ESTIMATEScore were elevated, while BMS-754807, ZM447439, JQ1, and NU7441 displayed increased sensitivity (Carboni et al., 2009; Kaestner et al., 2009; Ding et al., 2020). In experimental studies, these potential drugs have shown inhibitory effects on colorectal cancer, but their clinical safety and efficacy still need further validation. This study further confirmed the effectiveness of the scoring model in terms of immune infiltration and checkpoints; irrespective of microbiome-related tumor subtypes or microbiome-based risk scoring models, they exhibit excellent predictive value in the prognosis, immunotherapy, and drug sensitivity response of patients with colorectal cancer. This research further highlights the diverse functions and efficacy of the gut microbiome in colorectal cancer, such as its ability to predict patient outcomes, control immune responses in the intestines, affect the metabolism of carcinogens, influence the efficacy of immunotherapy, and regulate the environment surrounding tumors. Our findings align with the conclusions reported in various other studies (Hanus et al., 2021; Kim and Lee, 2021; Dougherty and Jobin, 2023; Yu et al., 2023; Zheng et al., 2023).

In this study, we explored the association between the gut microbiome and colorectal cancer, unveiling significant variations in microbiome compositions that correlate with distinct tumor subtypes. Our findings underscore the potential clinical applications of microbial biomarkers, as exemplified by genera such as Robiginitomaculum and Myxococcus, which are prevalent in the subtype associated with favorable prognosis, and Sutterella and Zymomonas, which dominate in the subtype linked to poor outcomes. This differential abundance not only highlights the pivotal role of microbial markers in influencing tumor progression and patient survival but also sets the stage for developing targeted therapeutic strategies that harness specific microbial profiles. Moreover, the identification and functional analysis of these biomarkers are crucial for refining existing treatment modalities and crafting novel personalized therapies. By analyzing the expression patterns of these markers across tumor subtypes, we can enhance the precision of patient-specific treatment predictions, thereby optimizing therapeutic outcomes. Additionally, these insights provide a new perspective on the complex interactions between the microbiome and the tumor immune microenvironment.

Although our research presents convincing proof of the intricate connection between colorectal cancer, the microbiome, and the immune environment, some constraints still persisted. The microbiome information was collected from openly accessible repositories, with a restricted number of samples. Moreover, the source and collection techniques of microbiome data may have a confounding effect on the analysis results. Additionally, although the sample information provided by the dataset used in this study is rich, it lacks information on directly isolated microbial samples. Future research should consider obtaining more accurate microbiome data from *in situ* tumor tissues. Additional research is also required to assess the impact of the identified microbial populations and microbiome communities on tumor advancement in terms of influencing immune responses, drug processing, and direct interactions with host cells.

In summary, our comprehensive analysis of the colorectal cancer microbiome, immune microenvironment, drug sensitivity, and other factors enhances our understanding of the multidimensional interactions in colorectal cancer biology as well as provides important clinical indications for the future improvements in the treatment of colorectal cancer. These findings offer a new perspective on treating colorectal cancer and suggest the possibility of improving patient treatment responses and long-term prognosis by modulating the microbiome composition or utilizing biomarkers presented by the microbiome.

## Data Availability

The original contributions presented in the study are included in the article/supplementary material. Further inquiries can be directed to the corresponding authors.

## Glossary

APC: Adenomatous Polyposis Coli
AUC: Area Under the Curve
CIBERSORTx: Cell-type Identification By Estimating Relative Subsets of RNA Transcripts
DC: Dendritic Cells
GEP: Gene Expression Profile
GSEA: Gene Set Enrichment Analysis
HRD: Homologous Recombination Defects
ICIs: Immune Checkpoint Inhibitors
KEGG: Kyoto Encyclopedia of Genes and Genomes
LASSO: Least Absolute Shrinkage and Selection Operator
MATH: Mutant-Allele Tumor Heterogeneity
PAC: Proportion of Ambiguous Clustering
PCA: Principal Component Analysis
ROC: Receiver Operating Characteristic
ssGSEA: Single Sample Gene Set Enrichment Analysis
TCGA: The Cancer Genome Atlas
TIDE: Tumor Immune Dysfunction and Exclusion
TMB: Tumor Mutation Burden
TME: Tumor Microenvironment
TPM: Transcripts Per Million
UMAP: Uniform Manifold Approximation and Projection
